# Effect of Eight-Month Exercise Intervention on Bone Outcomes of Young Opioid-Dependent Women

**DOI:** 10.3390/ijerph182111336

**Published:** 2021-10-28

**Authors:** Zenghui Ding, Zuchang Ma, Xianjun Yang, Yining Sun

**Affiliations:** Center for Information Technology of Sports and Health, Institute of Intelligent Machines, Hefei Institutes of Physical Science, Chinese Academy of Sciences , Hefei 230031, China; zhding_gz@126.com (Z.M.); xjyang@iim.ac.cn (X.Y.); ynsun@iim.ac.cn (Y.S.)

**Keywords:** drug abuse, exercise intervention, bone quality, body composition

## Abstract

Objective: To evaluate the bone response to an 8 month aerobic gymnastics training program in young opioid-addicted women. Design: Randomized controlled trial (parallel design). Setting: Women’s Specific Drug Rehabilitation Center in China. Patients: One hundred and two young women with low bone quality and previous opioid addiction were divided into two groups: (a) the low bone quality intervention experimental group (n = 55; age: 30.3 ± 6.1) and (b) the low bone quality observed control group (observation group; n = 47; age: 29.0 ± 5.3). Interventions: The intervention group took aerobic gymnastics regularly for 80 min/d and 5 d/wk for 8 months and completed follow-up testing. Main Outcome Measures: Substance use history and other life habits affecting bone quality were assessed by questionnaire-based interviews. Bone quality (stiffness-index, T-score, Z-score) was examined with quantitative ultrasound. Anthropometric characteristics (body weight, fat-free mass, fat mass) were obtained by bioelectrical impedance analysis. Results: After the 8 month intervention, the stiffness index of bone quality increased significantly (before: 82 ± 6, after: 108 ± 14, *p* < 0.05) in the experimental group. However, the bone quality did not change significantly in the controls (before: 79 ± 10, after: 77 ± 13, *p* > 0.05). The bone change in the difference group was significant (experimental group: 31.7% vs observation group: -0.03%). Fat mass decreased in the experimental group (experimental group: before: 19.6 ± 3.7 kg, after: 18.8 ± 4.0 kg, *p* < 0.05). Meanwhile, the change in fat-free mass was the determination of the change in bone quality in the experimental group. Conclusions: Our results suggested that aerobic gymnastics intervention can be an effective strategy for the prevention and treatment of drug-induced osteoporosis in detoxification addicts.

## 1. Background

Long-term opioid dependence in young people leads to drug-induced osteoporosis [1]. Long-term drug use probably affects bone metabolism, reduces trabecular bone mass, and suppresses hypothalamic secretion of gonadotropin-releasing hormone, consequently decreasing the level of gonadal hormones and leading to low bone quality and later-life osteoporosis [1,2]. Osteoporosis is an age-related disease characterized by a progressive loss of bone quality and microarchitectural deterioration, predisposing patients to fracture after minimal trauma or fall [3]. Although osteoporosis is typically thought to develop as a result of normal age-related losses in bone, persons who fail to attain their maximum peak bone mass during critical growing years may increase their risk of having osteoporosis [4]. Kay et al. reported that substance abuse in women yielded a higher risk of a variety of health problems than substance abuse in men [5]. Pharmacological therapy is the main option for osteoporosis, but the safety of long-term treatment is uncertain, especially for drug-induced osteoporosis patients. For example, estrogens, or hormonal substitutive treatment (HST), treatment using anti-resorptive agents, has potentially serious effects, such as breast or lung cancer, gallbladder disease, coronary events, stroke and venous thrombo-embolism. PTH1-34 (teriparatide), a drug that stimulates bone formation, causes potentially serious reactions, such as hypercholesterolemia, depression, dizziness, headache, sciatica, vertigo, palpitations, hypotension, dyspnea, nausea, vomiting, muscular cramps, asthenia and pruritus [6]. 

Exercise intervention could be an important means of prevention and treatment of drug-induced osteoporosis. Exercise for optimizing peak bone mass in young women is beneficial in old age [7]. Bone is inherently mechanosensitive, responding and adapting to its mechanical environment [8]. Regular exercise has effects on bone density, size and shape, resulting in substantial improvements in mechanical strength [9]. It is well known that high-impact exercise can enhance bone health in premenopausal women [10], but high-intensity exercise creates more exercise risks to frail people, and treatment options should be appropriate to their specific situation [11]. Aerobic exercise was also reported to benefit bone health by significantly reducing bone resorption activity [12,13]. Few studies have focused on the effects of aerobic training on drug-induced osteoporosis, fat-free mass (FFM) and fat mass (FM) in young opioid-addicted women. These questions merit further investigation.

In addition, body composition has been reported to be an important determinant of bone quality [14]. Many reports have shown that FFM has a positive relationship with bone quality [15,16]. However, it is uncertain whether changes in FFM determine changes in bone quality. We proposed the research hypothesis that increasing the degree of FFM through a special physical training program could be an effective treatment for improving the bone health of women with drug addiction in our previous study [1].

With a cohort of young opioid-dependent women, the aim of our study was to evaluate the effect of an 8 month aerobic gymnastics training program on bone quality and body composition in young opioid-dependent women so that suitable and effective nonpharmacotherapy intervention strategies could be employed to improve the bone health of young opioid-addicted women.

## 2. Methods

### 2.1. Design, Participants, Recruitment and Experimental Process

#### 2.1.1. Study Design

**Figure 1:**
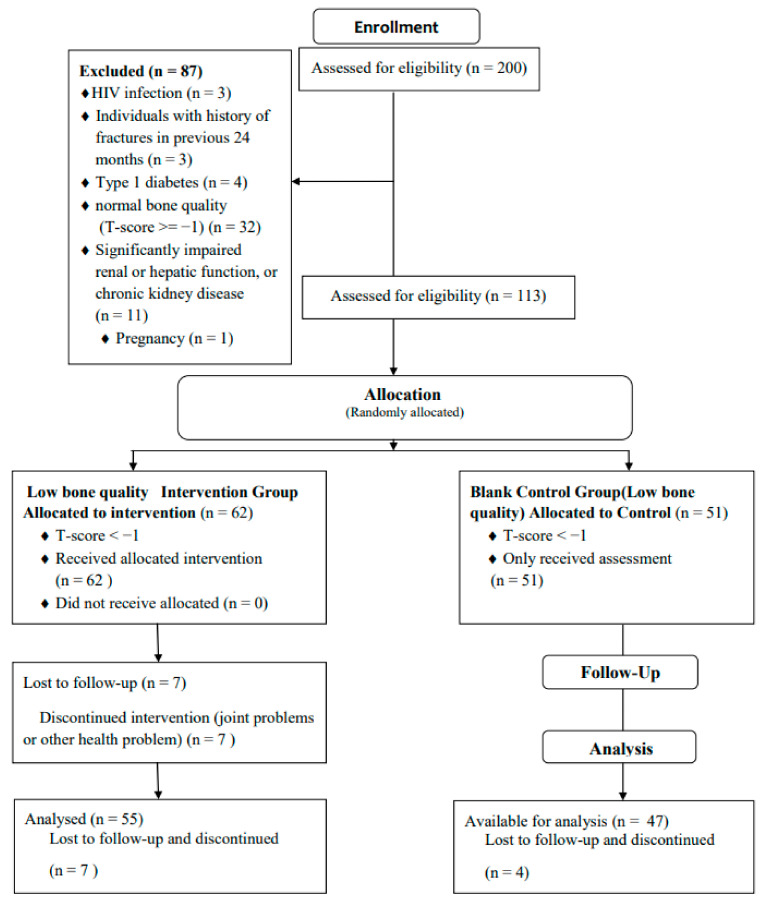
Flow chart of this research.

This was a randomized controlled trial study of long-term opioid-dependent women living in the middle area of China. The experimental process and quality control are shown in the experimental flowchart in Figure 1. Two-hundred subjects were recruited from the Women’s Specific Drug Rehabilitation Center of Anhui Province between May 2019 and August 2019. The recruitment criteria were as follows: (1) previous drug consumption > 3 years; (2) newcomers (within 4 months); and (3) age 20–40 years. They participated in a two-year voluntary isolation detoxification.

#### 2.1.2. Exclusion Criteria

The participants underwent questionnaire-based interviews and medical examinations. The exclusion criteria reduced the number of potential participants to 146. The exclusion criteria were as follows: (1) significantly impaired renal or hepatic function or chronic kidney disease; (2) a history of fractures in the previous 24 months; (3) type 1 diabetes; (4) HIV infection; (5) pregnancy due to altered hormonal balance [1,6] normal bone quality (T-score ≥ −1). Patients refusing to follow up in our study or who withdrew consent were also excluded. Finally, according to the conducted power calculation, a total of 113 patients aged 20–40 years were recruited [1].

#### 2.1.3. Experimental Process

Before the training program, measurements of the anthropometric characteristics and bone quality of all the participants were performed. Next, the samples were randomly divided in a double-blind experiment into two groups according to the odd even number of the last digit of the participants’ birthdays: (a) the low bone quality intervention experimental group (osteopenia or osteoporosis) (T-score < −1; n = 62) and (b) the low bone quality observed control group (observation group) (T-score < −1; n = 51). The patients in the experimental group underwent 8 months of aerobic gymnastics training from 5 August 2019 to 5 April 2020 and had their body composition and bone quality measured. The patients in the observation group did not take part in the exercise intervention, but they took part in all the body composition and bone quality measurements. The nutrient intake during the training period of the two groups was investigated and managed by nutrition experts. These nutrition experts surveyed the diet of the two groups through questionnaires every week and provided dietary guidance so that the calcium intake of the two groups was adequate and at the same level. Finally, one hundred and two patients (experimental group: n = 55, and observation group: n = 47) attended the entire experimental process, and eleven people were lost to follow-up due to joint problems or other problems.

#### 2.1.4. Experimental Approval

The research was approved by the Research Ethics Committee of the Hefei Institutes of Physical Science, Chinese Academy of Sciences. The RCT Registration number is ChiCTR1900021762. All participants provided informed consent, and the study was conducted in accordance with the guidelines of the Institute and Intelligence of Machines, Chinese Academy of Science.

### 2.2. Questionnaires—Background Characteristics

The questionnaire-based interviews were designed to obtain comprehensive information about all the participants. The questionnaires included four parts: medical history, history of drug use, smoking history, and nutritional calcium intake, as shown in Table 1.

To ensure the accuracy of the survey results, comparisons were made between the patients’ answers and the medical examination and official data, and the discrepancy was confirmed by the individuals.

### 2.3. Measurements of Anthropometric Characteristics

Body height (BH) was measured to the nearest 0.1 cm using a stadiometer (GMCS-I, XinDongHuaTeng Corp, Beijing, China). Body weight, FFM and fat mass (FM) were measured by a bioelectrical impedance analyzer (BX-BCA-100, Broshare Technology Corp., Hefei, China) [1], and the REG. NO. in the China Food and Drug Administration (CFDA) is 2210038. To eliminate the effects of diuretics, alcohol, intense exercise and fluids on the measurement, subjects emptied their bladder 30 min before the bioelectric impedance analysis (BIA) measurement. Subjects stood on bare feet with the heel and toe of each foot in contact with metal footpads, while lightly holding the two analyzer handgrips [1]. The coefficient of variance (CV) of the impedance measure was 0.4%. The values obtained from the BIA were supported by skinfold measurements using Harpenden calipers [1].

### 2.4. Measurements of Bone Quality

Bone quality includes a hierarchy of properties, such as bone densitometry and bone microarchitecture. Clinical studies showed that quantitative ultrasound (QUS) can be an effective means of estimating BMD [17] and assessing the effect of exercise intervention on bone [18]. In this study, bone quality was measured by a QUS device (BX-BDI-500A, Broshare Technology Corp., Hefei, China) that had been verified by clinical experiments with the REG. No. 20152230048 in CFDA. Speed of sound (SOS; m/s) and broadband ultrasound attenuation (BUA; dB/MHz) were measured on the right calcaneus. The stiffness index (SI), which has a lower precision error than either SOS or BUA alone, is calculated by the system according to the following formula: SI = 0.67 × BUA+0.28 × SOS −420 [19]. The SI provided information on bone structural parameters in addition to BMD, and a higher SI value indicated better bone health. SOS, BUA and SI have become common indexes in the assessment of bone health [20]. In addition, the values were also calculated as the T-score and Z-score, which were generated based on SI in the QUS device [21].

The bone quality of all the participants was diagnosed as normal (T-score ≥ −1.0), osteopenia (−2.5 ≤ T-score < −1.0), or osteoporosis (T-score < −2.5). The measurement took 5 min for each subject. To ensure the precision of the QUS measurements, each of the 10 subjects aged between 20 and 40 years received 20 measurements before every measurement. The values for the SOS, BUA and SI varied in the ranges of 1489–1623 m/s, 42–133 dB/MHz and 62–130, respectively, and the respective standard deviations were between 4 and 11 m/s, 1 and 5 dB/MHz and 1 and 4. The calculated coefficients of variation were 0.4%, 2.0% and 2.5% for the SOS, BUA and SI, respectively.

### 2.5. Aerobic Gymnastics Protocol

Two days after the initial 60 min trial, the experimental group began training 2 times (morning: 40 min, afternoon: 40 min) 5 days per week, completing an 8 month aerobic gymnastics exercise training program. The exercise training consisted of stretching and warm-up exercises (5 min); dynamic aerobic activities (30–40 min) involving stepping, skipping, graded walking, hopping, jogging, jumping, dancing and step choreographies; and cool-down/relaxation exercises (5 min). All the sessions were accompanied by appropriate music relevant to the required activity. The intensity of the main part of the training involved 40–60% of an individual’s maximal capacity and a Borg rating of perceived exertion scale (RPE) of 13–14, a level at which physical health benefits can be obtained. The warm-up exercises and the cool-down exercises involved 20–40% of an individual’s maximal capacity and a Borg RPE of 9–12. The training program was guided and corrected by a coach, and the intensity of exercise was also investigated by Borg RPE after every day’s training program [22]. Meanwhile, dynamic aerobic activities, such as the core exercise intervention, lasted for 30–40 min so that the body circulatory system could be fully mobilized. The observation group did not take part in any training programs. The physical activity level of the observation group was regularly investigated by questionnaire. Meanwhile, the observation group was available for all the examinations.

### 2.6. Statistical Analysis

Age, anthropometric characteristics, SI and T-score were expressed as the mean ± standard deviation. A *p* value of less than 0.05 was set as the level of significance. The student’s t-test was performed to compare the means and quantitative data between the low bone quality intervention experimental group and the low bone quality control group. *p* values, two-tailed, of <0.05 were considered statistically significant. A paired t-test was used to compare the values before and after the experiment. Independent t-tests were used to compare the experimental group and the control group. The association of the change in SI (∆SI) with each of the relevant factors (age, smoking status, body weight, FFM, FM, rate of fat mass (%Fat mass), SI, change in body weight (∆Body weight), change in fat-free mass (∆Fat-free mass), and change in fat mass (∆Fat mass)) was explored using linear regression. In a further step, the relationships between the outcomes and the factors of interest and potential determinants, including body weight, FFM, FM, %Fat mass, SI, ∆Body weight, ∆Fat-free mass, and ∆Fat mass, were investigated using multivariable linear regression. All the statistical analyses were conducted using SPSS for Windows, Version 22.0 (IBM Corp., Armonk, NY, USA).

## 3. Results

### 3.1. Baseline Characteristics of Participants

As listed in Table 2, the experimental group and observation group did not differ with regard to age, BH, BW, BMI, FFM, FM or bone quality (SI and T-Score). However, there was no difference in the type of drug use or method of drug use between the two groups. Meanwhile, in the course of the experiment, most of the samples in the two groups followed a well-balanced diet and a regular intake of calcium-rich foods.

### 3.2. Changes in Anthropometrics

There was a significant change in body composition in response to the exercise intervention (Table 3). Body weight and FM significantly decreased in the experimental group (*p* < 0.05; *p* < 0.05, respectively). Meanwhile, there were only significant decreases in BMI, fat mass index (FMI) and %FM in the experimental group. However, no significant changes in FFM and FFMI were observed in the observation group (Table 3).

### 3.3. Changes in Bone Quality

**Figure 2:**
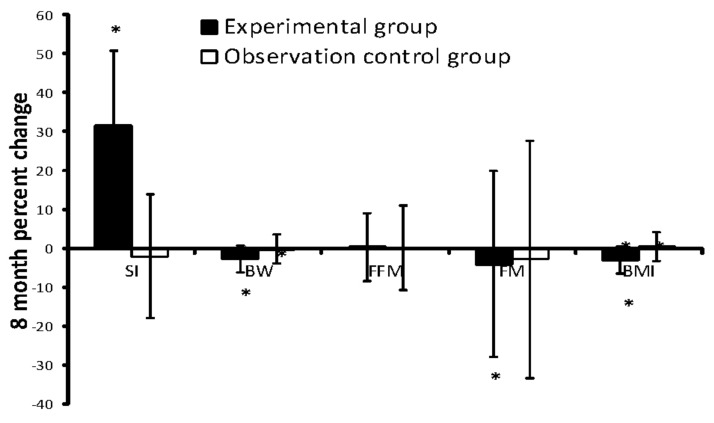
Mean difference (95% CI) after the 8 month exercise intervention in calcaneal bone quality and body composition in experimental group and observation control group. * *p* value determined by student’s t-test for differences in the basic characteristics of baseline and outcome, * *p* < 0.05; SI indicates stiffness index, SI = 0.67 × BUA + 0.28 × SOS − 420; BW indicates body weight; FFM indicates fat-free mass; FM indicates fat mass; BMI indicates body mass index.

After the 8 month exercise intervention, significant effects were observed for the SI (82 ± 6 vs 108 ± 14, *p* < 0.05) and the Z-score (−1.2 ± 0.4 VS 0.4 ± 0.9, *p* < 0.05) in the experimental group (Table 3), and the percentage of people with increasing bone quality was 100% (n = 55/55). The bone quality of the experimental group was significantly increased by 32.8% at the calcaneus (Table 3, Figure 2). However, no significant changes in SI were observed for the observation group (Table 3, Figure 2).

Table 4 shows the multivariate regression analysis for the prediction of bone quality in the experimental group. The relationships between the changes in calcaneal bone stiffness index (∆SI) and the factors of potential determinants (including body weight, FFM, FM, %fat mass, SI, ∆body weight, ∆fat-free mass, ∆fat mass) were investigated using multivariable linear regression. FFM and ∆FFM were positive predictors for SI (P = 0.010; *p* = 0.004, respectively), while SI and ∆body weight were negative predictors for ∆SI  (*p* = 0.016; *p* = 0.020, respectively).

## 4. Discussion

### 4.1. Main Findings

After 8 months of exercise interventions in young opioid-dependent women, we found that calcaneal bone quality was significantly increased and fat mass was significantly decreased in the experimental group, which was not found in the controls. Age is an important determinant of bone mass, but peak bone mass growth occurs mainly before the age of 20, and after the age of 20, especially after the age of 25, bone mass growth is not significant [23]. Through the design of the control groups, we demonstrated that the changes in bone and fat mass in the experimental group were not part of a natural recovery process. This study is novel because it is the first to demonstrate the efficacy of exercise-based interventions in increasing bone quality in young opioid women with low bone quality.

Data on the effect of exercise interventions on the bone quality of young opioid-dependent women are limited. We only found some cross-sectional studies that reported that effective physical activities were significantly lower in illicit drug abuse women than in healthy controls [1,24]. Furthermore, reports on the effect of aerobic interventions on young healthy women are controversial. Heinonen et al. [25] found no significant changes in BMD in growing girls after 9 months of step aerobics. By contrast, Friedlander et al. [26] reported that over a two-year period, a combined regimen of aerobics and weight training had beneficial effects on BMD and fitness parameters in young women. Wen et al. [22] reported that ten-week group-based step aerobics benefited bone metabolism and general health by significantly reducing bone resorption activity and improving functional fitness in women with low bone mass. Our results further demonstrated this point of view in young opioid-dependent women with low bone quality.

Compared with bone quality changes in the young opioid-dependent women in the experimental group, there were no significant changes in the observation group. These differences may have been due to the bone modeling and remodeling mechanisms of mechanical stress. Frost first described the mechanisms of “minimum effective strain” (MES), which predicts the time and the site of bone architecture changes as a result of adaptation to mechanical loads [27]. Strains below the MES are not considered to produce adaptive bone modeling, whereas those above it change bone architecture to reduce subsequent strains under load [27,28]. In our study, the experimental group took part in an 8 month aerobic gymnastics exercise training program. The exercise training consisted of: stretching and warm-up exercises (5 min); dynamic aerobic activities (30–40 min) involving stepping, skipping, graded walking, hopping, jogging, jumping, dancing and step choreographies; and cool-down/relaxation exercises (5 min). The warm-up exercises used enhance blood circulation, body temperature and muscle flexibility, and prepare the skeletal muscles, heart, and lungs for the more intense activity to come. The main part of the exercise training, including stepping, skipping, graded walking, hopping, jogging, jumping, dancing and step choreography exercises, may promote bone quality through the effect of direct mechanical loading on bone, which would activate osteoblasts and increase bone formation [29]. The cool-down/relaxation exercises used are conducive to the decomposition of lactic acid, reducing excessive stiffness and muscle tightening and improving muscle elasticity and are beneficial to bone health. The people in the observation group did not take part in any training program; therefore, the mechanical load on the bone did not change in their case, and their bone metabolism was not activated [30]. Meanwhile, bone turnover, the balance between bone resorption and bone formation, is influenced by other factors as well [31]. A decrease in the parathyroid hormone (PTH) determines reduced calcium absorption and, consequently, a reduction in BMD. Long-duration, low-intensity exercise was reported to raise PTH; thus, it is beneficial for bone health [32]. We suggested that the changes in bone in the exercise intervention group were not part of a natural recovery process [33] but a result of adaptation to mechanical loads.

In addition, some researchers have attempted to determine which duration and frequency of loading is most beneficial to the skeleton. Tsuji et al. reported that 3 months of aerobic exercise had no effect on bone quality, and they pointed out that the 3 month period may have been insufficient to stimulate osteogenesis [34]. The process of bone remodeling includes bone resorption and bone formation, and sufficient time is needed to complete the process [34]. Some studies have confirmed that exercise intervention for 6 months can improve bone quality [7]. In our study, considering the particularity of the research sample, the training period was set at 8 months. High-frequency/high-cycle-number exercise programs with low-to-moderate strain intensity were reported to have a positive effect on the bone strength of frail elderly individuals [35]. We confirmed that high-frequency/high-cycle-number exercise programs with low-to-moderate strain intensity for 8 months were beneficial to the bone health of young opioid-dependent women with low bone quality.

We also found that an 8 month aerobic gymnastics training program can be an effective strategy for the treatment of obesity in young opioid addicts undergoing rehabilitation. After drug withdrawal, drug addicts offset their dependence on drugs by consuming large amounts of food. Exercise intervention can be a healthy solution. Meanwhile, body composition is an important determinant of bone quality in general populations [36]. As shown in Table 4, our results further demonstrated that the changes in FFM determined changes in bone quality in the low bone quality intervention experimental group, which has seldom been reported in recent articles. Higher FFM leads to greater mechanical load on bone, which results in improved bone quality [29,30]. Meanwhile, as an important component of FFM, muscle contractions can produce mechanical stress, and mechanical stress on bone activates osteoblasts and increases bone formation [37].

### 4.2. Implications for the Treatment of Opioid Dependence

The findings of our study are of importance because they could be helpful for rehabilitation centers offering treatment programs. Aerobic training has been found to be a potential treatment for drug addiction, which may be related to its ability to facilitate dopaminergic transmission [38,39]. We further demonstrated that aerobic training was beneficial to the recovery of the bone quality of young opioid-addicted women. Increasing physician awareness of the effect of exercise intervention on the recovery of the bone quality of young opioid-addicted women will encourage non-pharmaceutical interventions to prevent or treat drug-induced osteoporosis and other problems.

### 4.3. Strengths and Limitations

The strengths of our study are that similar studies focusing on the effect of exercise intervention on young opioid-dependent women are scarce and, moreover, the experimental group and control groups were isolated in detoxification centers and participated in detoxification for two years. They had very similar living conditions and lived in the same macro-environment, and the training programs were well executed. However, our results should be interpreted in light of several limitations. First, other issues, such as serum concentrations of total testosterone, vitamin D level, serum concentrations of PTH, prolactin level, luteinizing hormone (LH) and sex hormone-binding globulin (SHBG), were not measured. The influences of these factors on bone were not analyzed. Second, we did not include measurements of bone status at other sites or use additional techniques, such as DXA. However, QUS measurement has become an important modality for the assessment of osteoporosis status [21].

## 5. Conclusions

In conclusion, our study indicated the positive effect of an 8 months aerobics exercise intervention on the recovery of bone quality in young opioid-dependent women. However, further research is needed to confirm these findings and to investigate whether such approaches could also be used by elderly individuals.

## Figures and Tables

**Table 1:** Questionnaires for the low bone quality intervention experimental group and low bone quality observation control group.

Questions	Data Type
**Medical History:**	
1. Individuals with history of fractures in previous 24 months	0 = No 1 = Yes
2. Pregnancy	0 = No 1 = Yes
3. Type 1 diabetes	0 = No 1 = Yes
4.Significantly impaired renal or hepatic function, or chronic kidney disease	0 = No 1 = Yes
**History of Drug Use (Patients only):**	
1.Type of drug use: (1) heroin, opium, morphine, and other opiates (2) cocaine (3) marijuana (4) amphetamines (5) hallucinogens (6) other drugs	Multi-selection
2. Age at onset of drug intake (year)	Quantitative
3. Duration of drug intake (years)	Quantitative
4. Frequency of drug use: (1) 3–7/week or more (3) 1–2/week (4) 1–2/month (5) seldom	Single-selection
5. Method of drug use: (1) injection (2) non-injection	Single-selection
6. Duration of methadone intake (weeks)	Quantitative
7. Daily methadone dose (mg)	Quantitative
8. Duration of drug intake (years)	Quantitative
**Smoking History:**	
(1) often (2) occasionally (3) seldom	Single-selection
**Nutritional Calcium Intake During the Training Period:**	
1.Diet: (1) well-balanced diet, regular intake of calcium-rich foods (2) occasional intake of calcium-rich foods (3) low nutritional calcium intake, rare intake of calcium-rich foods	Single-selection

**Table 2:** Baseline characteristics of the low bone quality intervention experimental group and low bone quality observation control group.

Variable	Experiment Group (n = 55)	Observation Group (n = 47)	*p*
Age(years)	30.3 ± 6.1	29.0 ± 5.3	0.332
**Anthropometrics**			
Body height (cm)	162 ± 4.9	162 ± 5.1	0.726
Body weight (kg)	62.7 ± 6.8	62.6 ± 6.4	0.993
Body mass index (kg/m^2^)	24.1 ± 2.4	24.0 ± 2.4	0.520
Fat Free mass (kg)	44.0 ± 2.9	42.5 ± 3.1	0.126
Fat mass (kg)	19.6 ± 3.7	20.0 ± 4.4	0.840
**Drug history**			
Heroin, opium, morphine, and other opiates (% of n)	87%	86%	0.482
Cocaine and other drugs (% of n)	73%	66%	0.115
Multiple substances (% of n)	53%	61%	0.183
High frequency of drug use ^a^	88%	89%	0.365
Age at onset of drug intake (year)	21.7 ± 5.4	21.5 ± 4.4	0.578
Injecting drug users (% of n)	17%	19%	0.135
Duration of drug intake (years)	8.6 ± 3.7	8.9±3.1	0.563
Smoking (% of n)	96%	94%	0.854
**Bone quality**			
SI ^b^	82 ± 6	79 ± 12	0.096
T-Score	−1.3 ± 0.3	−1.4 ± 0.8	0.121
**Nutrient intake status during the training period**			
Well-balanced diet, regular intake of calcium-rich foods. (% of n)	93.6%	95.2%	0.256
**Physical activity per day**			
Aerobic gymnastics time (min)	80	——	——

The data are shown as the mean ± standard deviation; ^a^ High frequency of drug use: 3–7/week or more; ^b^ SI indicates stiffness index, SI = 0.67 × BUA + 0.28 × SOS −420.

**Table 3:** Changes in anthropometric and bone quality.

Characteristic	Experiment Group (n = 55)			Observed Control Group (n = 47)			*p*-value
	Before	After	*p*_1_	Before	After	*p*_1_	*p*_2_
**Anthropometrics**							
Body weight (kg)	62.7 ± 6.8	60.9 ± 6.5 *^,†^	0.025	62.6 ± 6.4	62.5 ± 6.1	0.521	0.000
Fat-free mass (kg)	42.0 ± 2.9	42.2 ± 3.2	0.352	42.5 ± 3.1	42.9 ± 3.2	0.235	0.223
Fat mass (kg)	19.6 ± 3.7	18.8 ± 4.0 *^,†^	0.011	20.0 ± 4.4	19.8 ± 4.3	0.175	0.012
BMI (kg/m^2^)	24.1 ± 2.4	23.4 ± 2.3 *	0.016	24.0 ± 2.4	23.9 ± 2.3	0.156	0.071
FFMI (kg/m^2^)	16.9 ± 1.5	16.1 ± 0.7	0.265	16.3 ± 0.7	16.5 ± 0.7	0.235	0.131
FMI (kg/m^2^)	7.5 ± 1.4	7.2 ± 1.5 *^,†^	0.002	7.7 ± 1.8	7.4 ± 1.7	0.095	0.015
%FM	31.6 ± 6.3	29.7 ± 3.7 *	0.003	31.7 ± 4.4	30.8 ± 4.5	0.087	0.089
**Bone quality**							
SI	82 ± 6	108 ± 14 *^,†^	0.000	79 ± 10	77 ± 13	0.156	0.000
T-Score	−1.3 ± 0.3	0.2 ± 0.7 *^,†^	0.000	−1.4 ± 0.5	−1.5 ± 0.7	0.113	0.000
Z-Score	−1.3 ± 0.4	0.4 ± 0.9 *^,†^	0.000	−1.5 ± 0.5	−1.5 ± 0.8	0.189	0.000

The data are shown as the mean ± standard deviation; *p_1_* different from the same group before and after the experiment; * Significantly different from the same group before and after the experiment, *p* < 0.05; *p*_2_ different from the changes between the experiment and observed control group. ^†^ Significantly different from the changes between experiment and observed control group, *p* < 0.05; FFMI indicates fat-free mass index, fat-free mass /height squared; FMI indicates fat mass index, fat mass/height squared; %FM=FM/Body weight*100; SI indicates stiffness index, SI = 0.67 × BUA + 0.28 × SOS − 420.

**Table 4:** Multivariate regression analysis of changes in calcaneal bone quality in the low bone quality intervention experimental group.

Dependent Variable	Independent Variable	Standard β	*p*
		Low Bone Quality Intervention Experimental Group	
∆SI			
Model	Body weight	−4.390	0.077
	Fat-free mass	5.275	0.010
	Fat mass	0.765	0.562
	%Fat mass	0.103	0.936
	SI	−0.126	0.016
	∆Body weight	−0.992	0.020
	∆Fat-free mass	2.446	0.004
	∆Fat mass	0.800	0.314
	R = 0.744		

∆SI indicates changes in calcaneal bone stiffness index; SI indicates stiffness index before training program, SI = 0.67 × BUA+0.28 × SOS – 420; %Fat mass = FM/Body weight × 100; c) ∆body weight indicates changes in body weight; ∆fat-free mass indicates changes in fat-free mass; ∆fat mass indicates changes in fat mass.

## Abbreviations

BMI: body mass index; FFM: fat-free mass; FM: fat mass; QUS: quantitative ultrasound; BMD: bone mineral density; SOS: speed of sound (m/s); BUA: broadband ultrasound attenuation (dB/MHz); SI: bone stiffness index.

## References

[1] Ding Z., Chen Y., Wang X., Zhou X., Xu Y., Ma Z., Sun Y., Jiang M. (2017). A comparison of bone quality and its determinants in young opioid-dependent women with healthy control group. Drug Alcohol Depend..

[2] Gotthardt F., Huber C., Thierfelder C., Grize L., E Kraenzlin M., Scheidegger C., Meier C. (2016). Bone mineral density and its determinants in men with opioid dependence. J. Bone Miner. Metab..

[3] Harvey N., Dennison E., Cooper C. (2014). Osteoporosis: A Lifecourse Approach. J. Bone Miner. Res..

[4] Nordström P., Sievänen H., Gustafson Y., Pedersen N.L., Nordström A. (2013). High physical fitness in young adulthood reduces the risk of fractures later in life in men: A nationwide cohort study. J. Bone Miner. Res..

[5] Kay A., Taylor T.E., Barthwell A.G., Wichelecki J., Leopold V. (2010). Substance Use and Women’s Health. J. Addict. Dis..

[6] Iolascon G., Moretti A., Toro G., Gimigliano F., Liguori S., Paoletta M. (2020). Pharmacological Therapy of Osteoporosis: What’s New?. Clin. Interv. Aging.

[7] Bailey C.A., Brooke-Wavell K. (2008). Exercise for optimising peak bone mass in women. Proc. Nutr. Soc..

[8] Mellon S.J., Tanner K.E. (2012). Bone and its adaptation to mechanical loading: A review. Int. Mater. Rev..

[9] Tveit M., Rosengren B.E., Nilsson J.Å., Karlsson M.K. (2015). Exercise in youth: High bone mass, large bone size, and low fracture risk in old age. Scand J. Med. Sci Spor..

[10] Carter M.I., Hinton P.S. (2014). Physical activity and bone health. Mo. Med..

[11] Senderovich H., Kosmopoulos A. (2018). An Insight into the Effect of Exercises on the Prevention of Osteoporosis and Associated Fractures in High-risk Individuals. Rambam Maimonides Med. J..

[12] Wen H.J., Huang T.H., Li T.L., Chong P.N., Ang B.S. (2016). Effects of short-term step aerobics exercise on bone metabolism and functional fitness in postmenopausal women with low bone mass. Osteoporosis Int..

[13] Ayudthaya W.C.N., Kritpet T. (2015). Effects of Low Impact Aerobic Dance and Fitball Training on Bone Resorption and Health-Related Physical Fitness in Thai Working Women. J. Med. Assoc. Thail..

[14] Ding Z., Chen Y., Xu Y., Zhou X., Xu Y., Ma Z., Sun Y. (2016). Impact of Age, Gender, and Body Composition on Bone Quality in an Adult Population From the Middle Areas of China. J. Clin. Densitom..

[15] Wang M.C., Bachrach L.K., Van Loan M., Hudes M., Flegal K.M., Crawford P.B. (2005). The relative contributions of lean tissue mass and fat mass to bone density in young women. Bone.

[16] Bakker I., Twisk J.W.R., van Mechelen W., Kemper H.C.G. (2003). Fat-free body mass is the most important body composition determinant of 10-yr longitudinal development of lumbar bone in adult men and women. J. Clin. Endocrinol. Metab..

[17] Ana T.C., Dimitris V., Esther U.G., Asuncion F.M., Ivan C.R., Vicente M.V., Luis G.M. (2018). Agreement Between Dual-Energy X-Ray Absorptiometry and Quantitative Ultrasound to Evaluate Bone Health in Adolescents: The PRO-BONE Study. Pediatric Exerc..

[18] Kurosaka S., Ueda T., Deguchi T., Okihara K., Yuzaki Y. (2019). Effects of the Building Osteo Neatly Exercise (BONE) program on quantitative ultrasound parameters and plantar pressure distribution in college-aged women. J. Ence Med. Sport.

[19] Njeh C.F., Boivin C.M., Langton C.M. (1997). The role of ultrasound in the assessment of osteoporosis: A review. Osteoporosis Int..

[20] Lejla K., Stephanie Z., Matthias N., Henry V., Nele F., Anke H. (2018). Associations Between Plasma Chemerin Concentrations and Bone Quality in Adults From the General Population. Endocrinology.

[21] Liu J.-M., Ma L.-Y., Bi Y.-F., Xu Y., Huang Y., Xu M., Zhao H.-Y., Sun L.-H., Tao B., Li X.-Y. (2012). A Population-Based Study Examining Calcaneus Quantitative Ultrasound and Its Optimal Cut-Points to Discriminate Osteoporotic Fractures among 9352 Chinese Women and Men. J. Clin. Endocrinol. Metab..

[22] Wen H.J., Huang T.H., Li T.L., Chong P.N., Ang B.S. (2017). Effects of short-term step aerobics exercise on bone metabolism and functional fitness in postmenopausal women with low bone mass. Osteoporosis Int..

[23] Weaver C.M., Gordon C.M., Janz K.F., Kalkwarf H.J., Lappe J.M., Lewis R., Karma M.O., Wallace T.C., Zemel B.S. (2016). The National Osteoporosis Foundation’s position statement on peak bone mass development and lifestyle factors: A systematic review and implementation recommendations. Osteoporosis Int..

[24] Milos G., Gallo L.M., Sosic B., Uebelhart D., Goerres G., Haeuselmann H.J., Eich D. (2011). Bone Mineral Density in Young Women on Methadone Substitution. Calcif. Tissue Int..

[25] Heinonen A., Sievänen H., Kannus P., Oja P., Pasanen M., Vuori I. (2000). High-Impact Exercise and Bones of Growing Girls: A 9-Month Controlled Trial. Osteoporosis Int..

[26] Friedlander A.L., Genant H.K., Sadowsky S., Byl N.N., Glüer C.C. (1995). A two-year program of aerobics and weight training enhances bone mineral density of young women. J. Bone Miner. Res. Off. J. Am. Soc. Bone Miner. Res..

[27] Frost H.M. (1987). Bone “mass” and the “mechanostat”. A Proposal Anat Rec..

[28] Frost H.M. (2003). Bone’s mechanostat: A 2003 update. Anat. Rec. Part. A Discov. Mol. Cell. Evol. Biol..

[29] Ehrlich P.J., Lanyon L.E. (2002). Mechanical strain and bone cell function: A review. Osteoporosis Int..

[30] Rodan G.A. (1997). Bone mass homeostasis and bisphosphonate action. Bone.

[31] Codrea C.I., Croitoru A.-M., Baciu C.C., Melinescu A., Ficai D., Fruth V., Ficai A. (2021). Advances in Osteoporotic Bone Tissue Engineering. J. Clin. Med..

[32] Ljunghall S., Joborn H., Roxin L.-E., Rastad J., Wide L., Åkerström G. (2010). Prolonged low-intensity exercise raises the serum parathyroid hormone levels. Clin. Endocrinol..

[33] Gleeson P.B., Protas E.J., Leblanc A.D., Schneider V.S., Evans H.J. (1990). Effects of weight lifting on bone mineral density in premenopausal women. J. Bone Miner. Res. Off. J. Am. Soc. Bone Miner. Res..

[34] Tsuji S., Haneda M., Lee C., Katsukawa F., Onishi S., Yamazaki H. (1996). Effects of 3 months of non-weight-bearing cycle ergometric exercise on bone metabolism. J. Bone Miner. Metab..

[35] Kemmler W., Von S.S. (2011). Exercise and osteoporosis-related fractures: Perspectives and recommendations of the sports and exercise scientist. Physician Sportsmed..

[36] Felson D.T., Zhang Y.Q., Hannan M.T., Anderson J.J. (1993). Effects Of Weight And Body-Mass Index On Bone-Mineral Density In Men And Women—The Framingham-Study. J. Bone Miner. Res..

[37] Schiessl H., Frost H.M., Jee W.S.S. (1998). Estrogen and bone-muscle strength and mass relationships. Bone.

[38] Smith M.A., Schmidt K.T., Iordanou J.C., Mustroph M.L. (2008). Aerobic exercise decreases the positive-reinforcing effects of cocaine. Drug Alcohol Depend..

[39] Lynch W.J., Peterson A.B., Sanchez V., Abel J., Smith M.A. (2013). Exercise as a novel treatment for drug addiction: A neurobiological and stage-dependent hypothesis. Neurosci. Biobehav. Rev..

